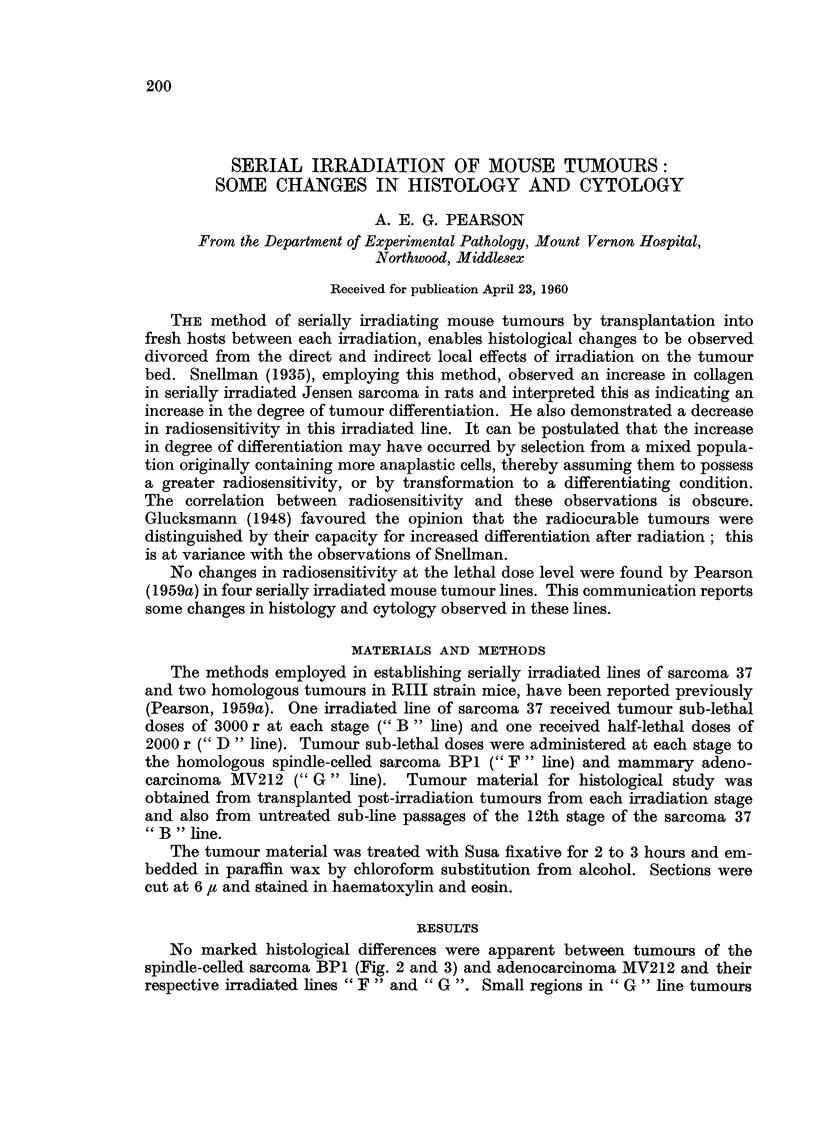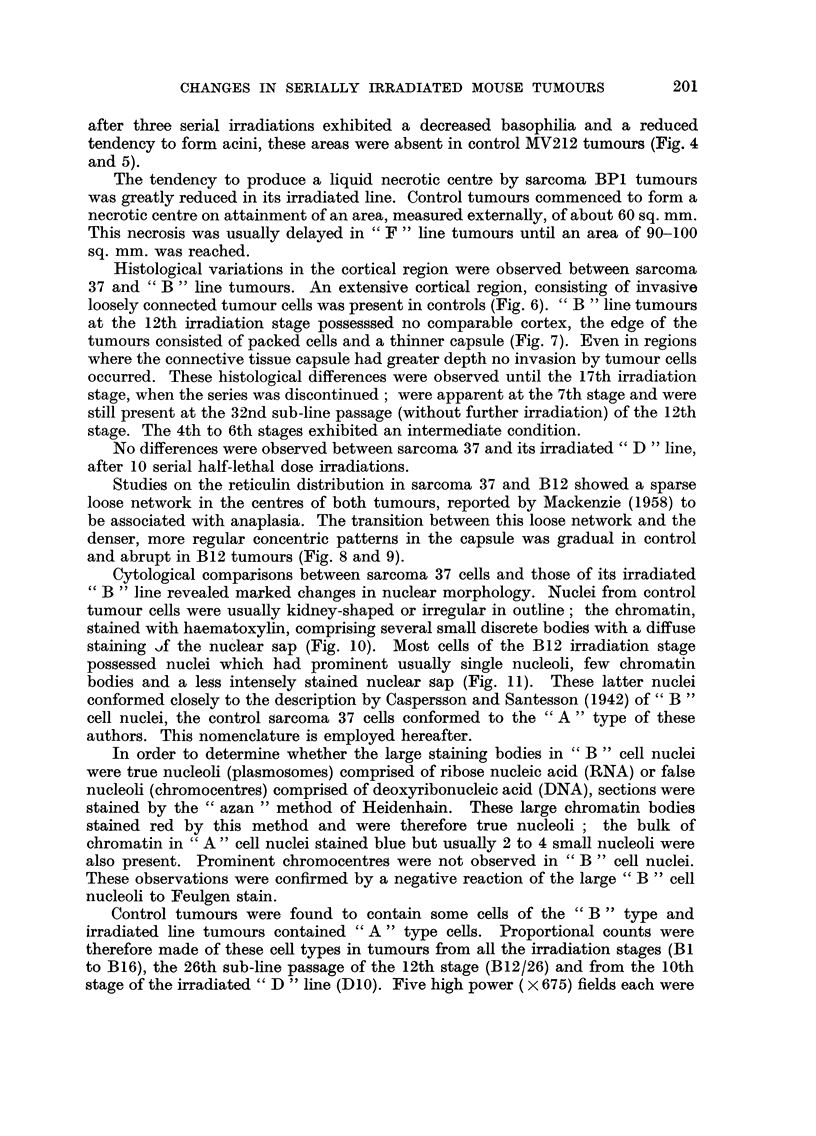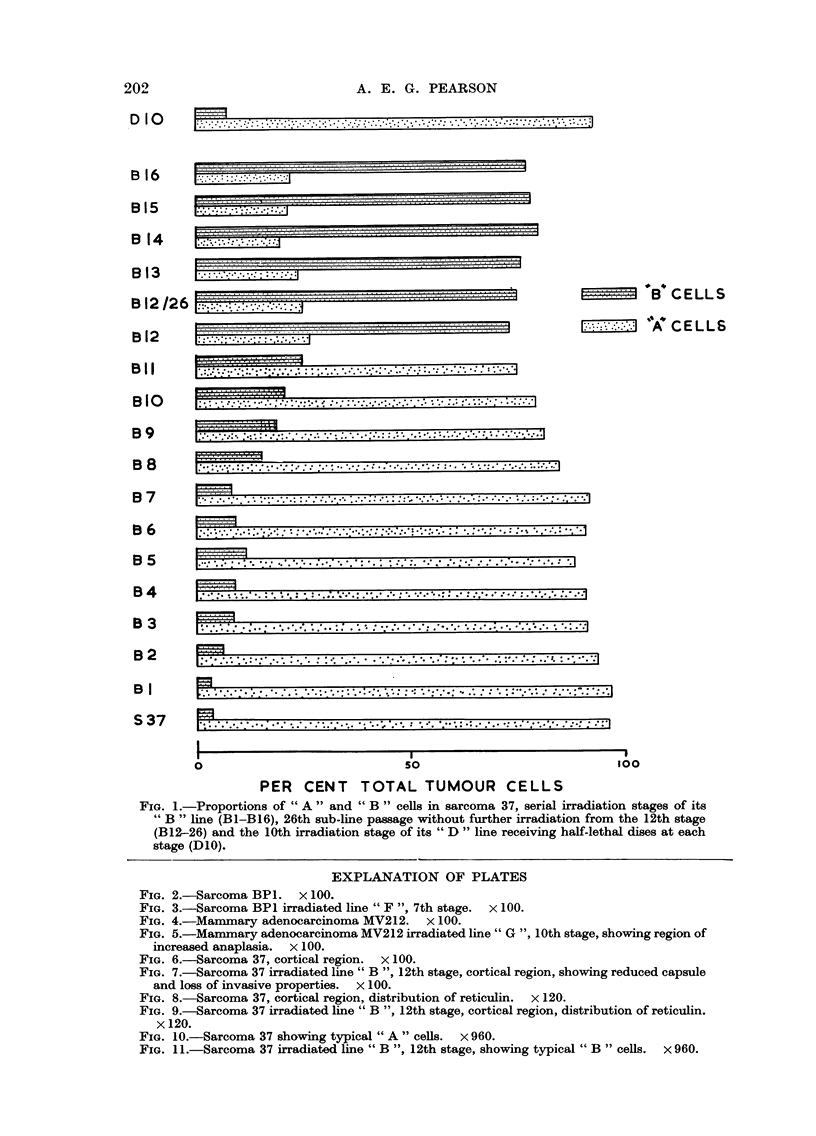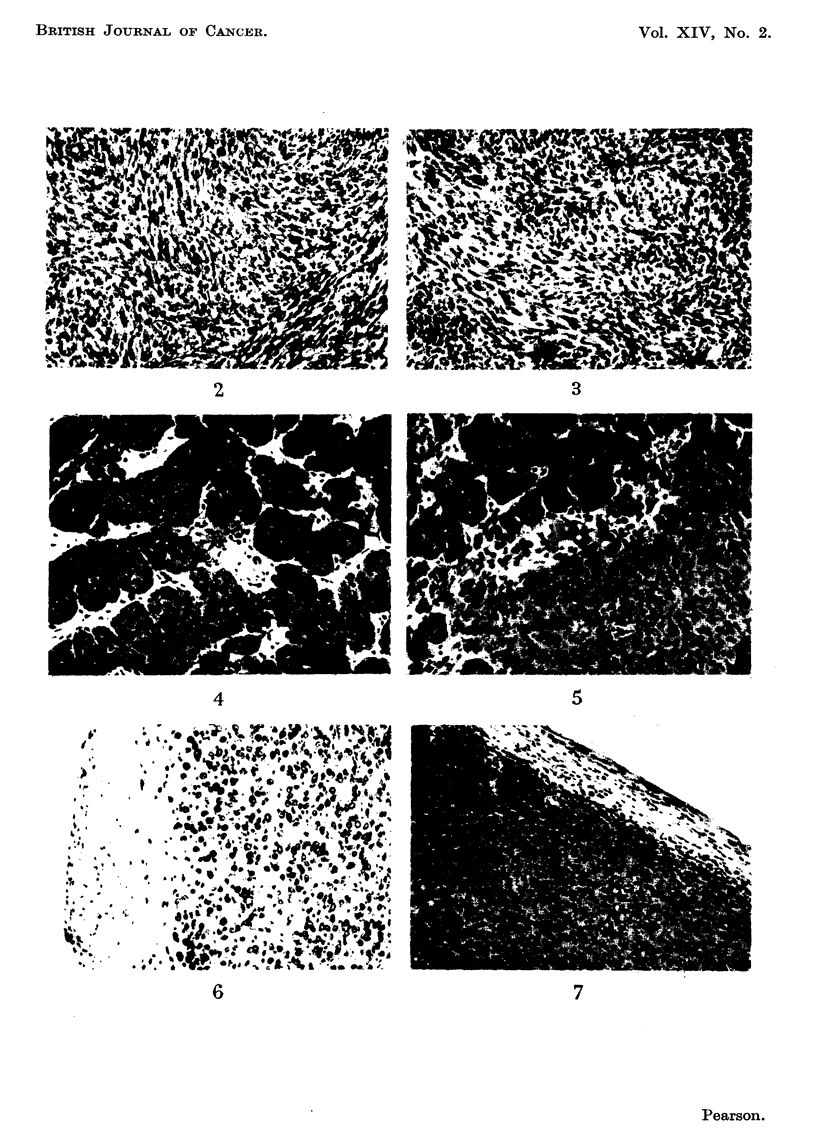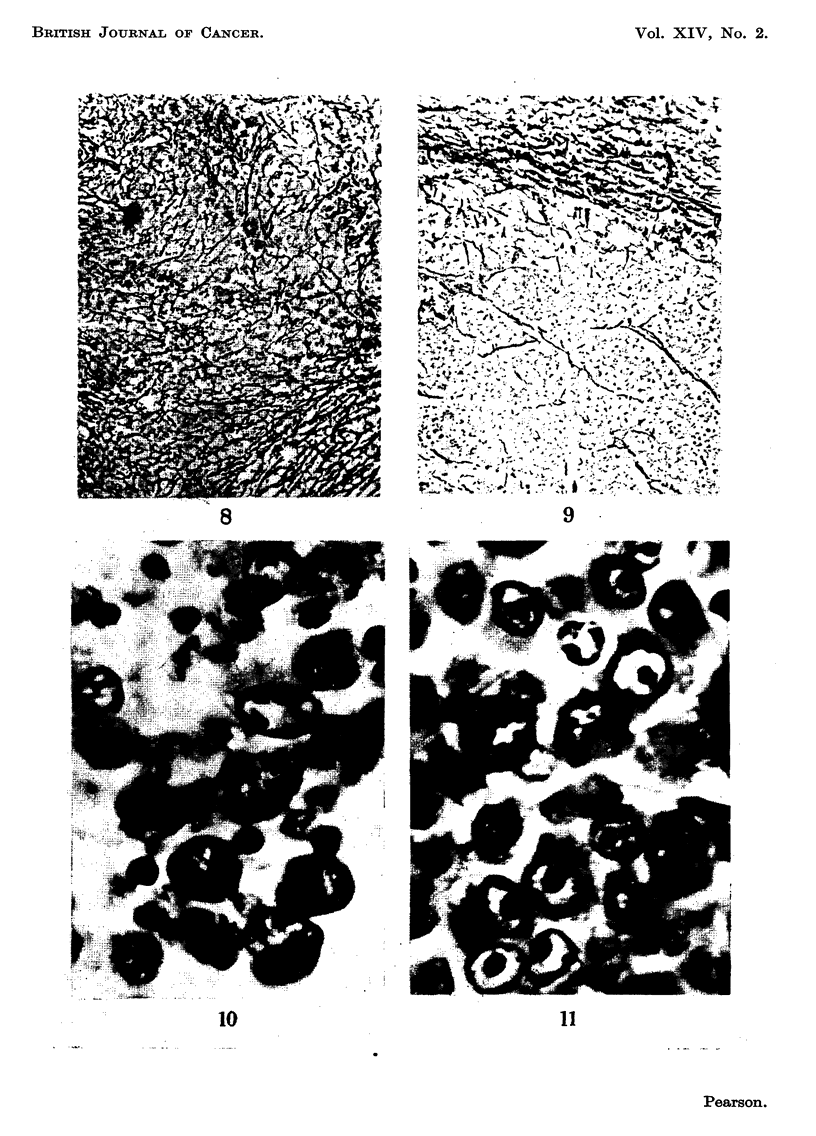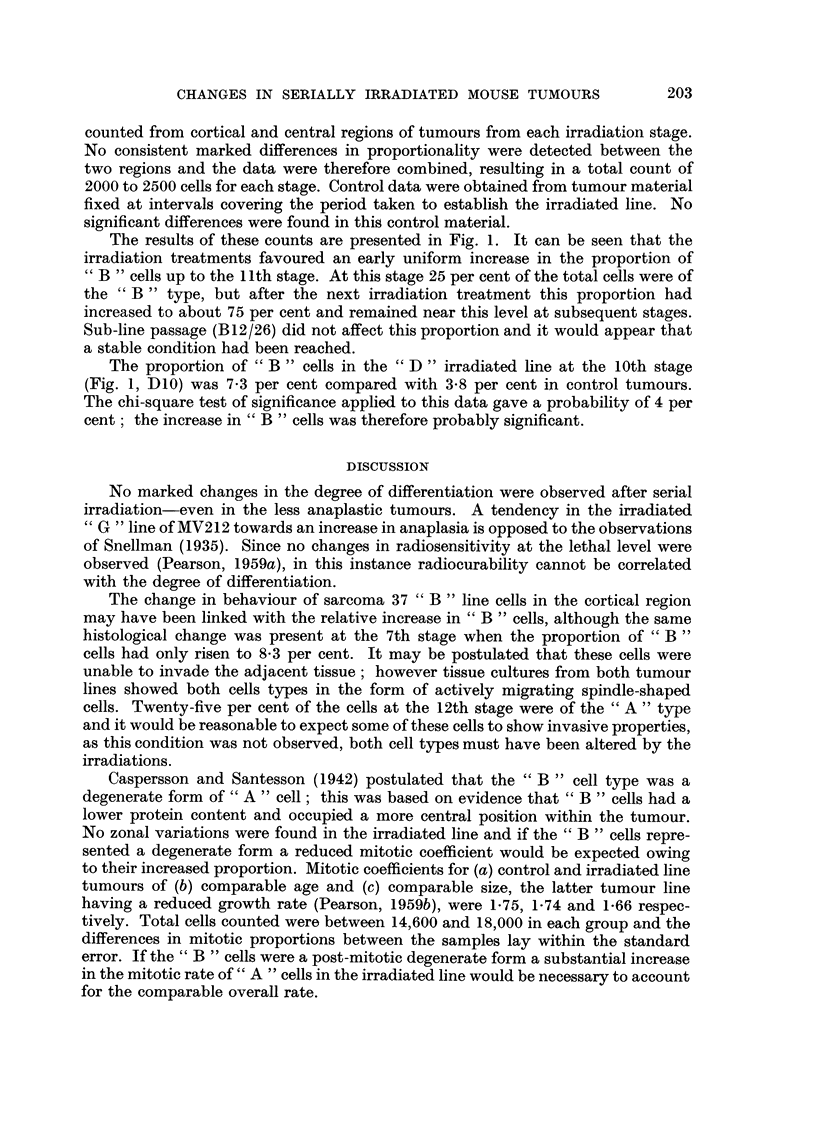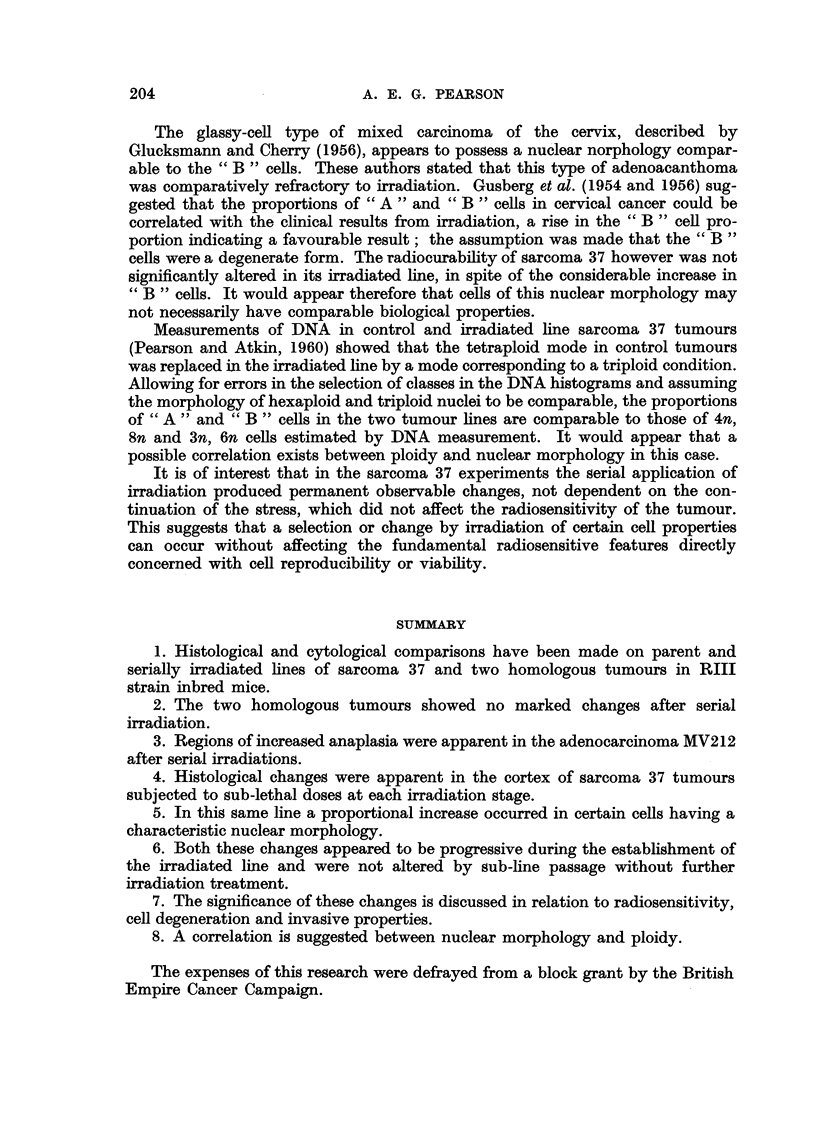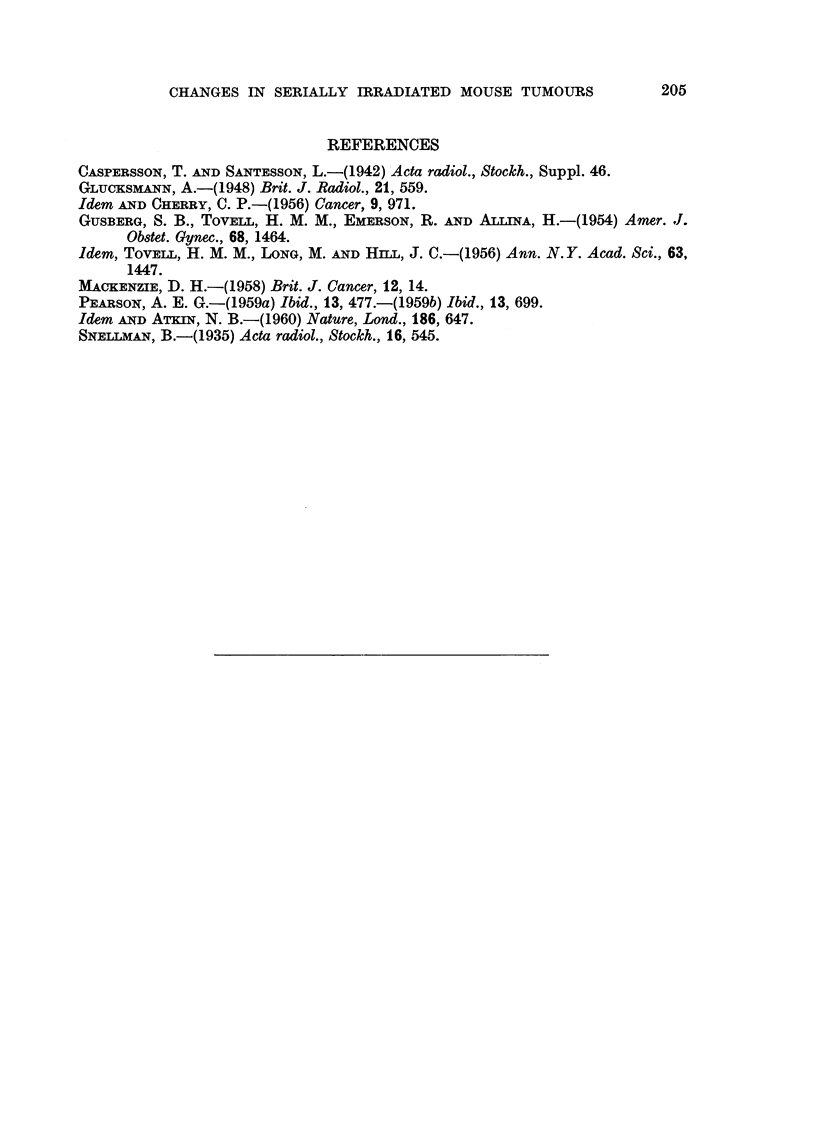# Serial Irradiation of Mouse Tumours: Some Changes in Histology and Cytology

**DOI:** 10.1038/bjc.1960.24

**Published:** 1960-06

**Authors:** A. E. G. Pearson

## Abstract

**Images:**


### SERIAL IRRADIATION OF MOUSE TUMOURS: SOME CHANGES IN HISTOLOGY AND CYTOLOGY

A. E. G. PEARSON

*From the Department of Experimental Pathology, Mount Vernon Hospital, Northwood, Middlesex*

Received for publication April 23, 1960

THE method of serially irradiating mouse tumours by transplantation into fresh hosts between each irradiation, enables histological changes to be observed divorced from the direct and indirect local effects of irradiation on the tumour bed. Snellman (1935), employing this method, observed an increase in collagen in serially irradiated Jensen sarcoma in rats and interpreted this as indicating an increase in the degree of tumour differentiation. He also demonstrated a decrease in radiosensitivity in this irradiated line. It can be postulated that the increase in degree of differentiation may have occurred by selection from a mixed population originally containing more anaplastic cells, thereby assuming them to possess a greater radiosensitivity, or by transformation to a differentiating condition. The correlation between radiosensitivity and these observations is obscure. Glucksmann (1948) favoured the opinion that the radiocurable tumours were distinguished by their capacity for increased differentiation after radiation; this is at variance with the observations of Snellman.

No changes in radiosensitivity at the lethal dose level were found by Pearson (1959*a*) in four serially irradiated mouse tumour lines. This communication reports some changes in histology and cytology observed in these lines.

### MATERIALS AND METHODS

The methods employed in establishing serially irradiated lines of sarcoma 37 and two homologous tumours in RIII strain mice, have been reported previously (Pearson, 1959*a*). One irradiated line of sarcoma 37 received tumour sub-lethal doses of 3000 r at each stage (" B " line) and one received half-lethal doses of 2000 r (" D " line). Tumour sub-lethal doses were administered at each stage to the homologous spindle-celled sarcoma BP1 (" F " line) and mammary adenocarcinoma MV212 (" G " line). Tumour material for histological study was obtained from transplanted post-irradiation tumours from each irradiation stage and also from untreated sub-line passages of the 12th stage of the sarcoma 37 " B " line.

The tumour material was treated with Susa fixative for 2 to 3 hours and embedded in paraffin wax by chloroform substitution from alcohol. Sections were cut at 6 $\mu$ and stained in haematoxylin and eosin.

### RESULTS

No marked histological differences were apparent between tumours of the spindle-celled sarcoma BP1 (Fig. 2 and 3) and adenocarcinoma MV212 and their respective irradiated lines " F " and " G ". Small regions in " G " line tumours after three serial irradiations exhibited a decreased basophilia and a reduced tendency to form acini, these areas were absent in control MV212 tumours (Fig. 4 and 5).

The tendency to produce a liquid necrotic centre by sarcoma BP1 tumours was greatly reduced in its irradiated line. Control tumours commenced to form a necrotic centre on attainment of an area, measured externally, of about 60 sq. mm. This necrosis was usually delayed in " F " line tumours until an area of 90–100 sq. mm. was reached.

Histological variations in the cortical region were observed between sarcoma 37 and " B " line tumours. An extensive cortical region, consisting of invasive loosely connected tumour cells was present in controls (Fig. 6). " B " line tumours at the 12th irradiation stage possesssed no comparable cortex, the edge of the tumours consisted of packed cells and a thinner capsule (Fig. 7). Even in regions where the connective tissue capsule had greater depth no invasion by tumour cells occurred. These histological differences were observed until the 17th irradiation stage, when the series was discontinued ; were apparent at the 7th stage and were still present at the 32nd sub-line passage (without further irradiation) of the 12th stage. The 4th to 6th stages exhibited an intermediate condition.

No differences were observed between sarcoma 37 and its irradiated " D " line, after 10 serial half-lethal dose irradiations.

Studies on the reticulin distribution in sarcoma 37 and B12 showed a sparse loose network in the centres of both tumours, reported by Mackenzie (1958) to be associated with anaplasia. The transition between this loose network and the denser, more regular concentric patterns in the capsule was gradual in control and abrupt in B12 tumours (Fig. 8 and 9).

Cytological comparisons between sarcoma 37 cells and those of its irradiated " B " line revealed marked changes in nuclear morphology. Nuclei from control tumour cells were usually kidney-shaped or irregular in outline ; the chromatin, stained with haematoxylin, comprising several small discrete bodies with a diffuse staining of the nuclear sap (Fig. 10). Most cells of the B12 irradiation stage possessed nuclei which had prominent usually single nucleoli, few chromatin bodies and a less intensely stained nuclear sap (Fig. 11). These latter nuclei conformed closely to the description by Caspersson and Santesson (1942) of " B " cell nuclei, the control sarcoma 37 cells conformed to the " A " type of these authors. This nomenclature is employed hereafter.

In order to determine whether the large staining bodies in " B " cell nuclei were true nucleoli (plasmosomes) comprised of ribose nucleic acid (RNA) or false nucleoli (chromocentres) comprised of deoxyribonucleic acid (DNA), sections were stained by the " azan " method of Heidenhain. These large chromatin bodies stained red by this method and were therefore true nucleoli ; the bulk of chromatin in " A " cell nuclei stained blue but usually 2 to 4 small nucleoli were also present. Prominent chromocentres were not observed in " B " cell nuclei. These observations were confirmed by a negative reaction of the large " B " cell nucleoli to Feulgen stain.

Control tumours were found to contain some cells of the " B " type and irradiated line tumours contained " A " type cells. Proportional counts were therefore made of these cell types in tumours from all the irradiation stages (B1 to B16), the 26th sub-line passage of the 12th stage (B12/26) and from the 10th stage of the irradiated " D " line (D10). Five high power ($\times 675$) fields each were

FIG. 1.—Proportions of " A " and " B " cells in sarcoma 37, serial irradiation stages of its " B " line (B1–B16), 26th sub-line passage without further irradiation from the 12th stage (B12–26) and the 10th irradiation stage of its " D " line receiving half-lethal dises at each stage (D10).

---

### EXPLANATION OF PLATES

FIG. 2.—Sarcoma BP1. ×100.
FIG. 3.—Sarcoma BP1 irradiated line " F ", 7th stage. ×100.
FIG. 4.—Mammary adenocarcinoma MV212. ×100.
FIG. 5.—Mammary adenocarcinoma MV212 irradiated line " G ", 10th stage, showing region of increased anaplasia. ×100.
FIG. 6.—Sarcoma 37, cortical region. ×100.
FIG. 7.—Sarcoma 37 irradiated line " B ", 12th stage, cortical region, showing reduced capsule and loss of invasive properties. ×100.
FIG. 8.—Sarcoma 37, cortical region, distribution of reticulin. ×120.
FIG. 9.—Sarcoma 37 irradiated line " B ", 12th stage, cortical region, distribution of reticulin. ×120.
FIG. 10.—Sarcoma 37 showing typical " A " cells. ×960.
FIG. 11.—Sarcoma 37 irradiated line " B ", 12th stage, showing typical " B " cells. ×960.

2

3

4

5

6

7

8

9

10

11

counted from cortical and central regions of tumours from each irradiation stage. No consistent marked differences in proportionality were detected between the two regions and the data were therefore combined, resulting in a total count of 2000 to 2500 cells for each stage. Control data were obtained from tumour material fixed at intervals covering the period taken to establish the irradiated line. No significant differences were found in this control material.

The results of these counts are presented in Fig. 1. It can be seen that the irradiation treatments favoured an early uniform increase in the proportion of " B " cells up to the 11th stage. At this stage 25 per cent of the total cells were of the " B " type, but after the next irradiation treatment this proportion had increased to about 75 per cent and remained near this level at subsequent stages. Sub-line passage (B12/26) did not affect this proportion and it would appear that a stable condition had been reached.

The proportion of " B " cells in the " D " irradiated line at the 10th stage (Fig. 1, D10) was 7·3 per cent compared with 3·8 per cent in control tumours. The chi-square test of significance applied to this data gave a probability of 4 per cent ; the increase in " B " cells was therefore probably significant.

### DISCUSSION

No marked changes in the degree of differentiation were observed after serial irradiation—even in the less anaplastic tumours. A tendency in the irradiated " G " line of MV212 towards an increase in anaplasia is opposed to the observations of Snellman (1935). Since no changes in radiosensitivity at the lethal level were observed (Pearson, 1959*a*), in this instance radiocurability cannot be correlated with the degree of differentiation.

The change in behaviour of sarcoma 37 " B " line cells in the cortical region may have been linked with the relative increase in " B " cells, although the same histological change was present at the 7th stage when the proportion of " B " cells had only risen to 8·3 per cent. It may be postulated that these cells were unable to invade the adjacent tissue ; however tissue cultures from both tumour lines showed both cells types in the form of actively migrating spindle-shaped cells. Twenty-five per cent of the cells at the 12th stage were of the " A " type and it would be reasonable to expect some of these cells to show invasive properties, as this condition was not observed, both cell types must have been altered by the irradiations.

Caspersson and Santesson (1942) postulated that the " B " cell type was a degenerate form of " A " cell ; this was based on evidence that " B " cells had a lower protein content and occupied a more central position within the tumour. No zonal variations were found in the irradiated line and if the " B " cells represented a degenerate form a reduced mitotic coefficient would be expected owing to their increased proportion. Mitotic coefficients for (*a*) control and irradiated line tumours of (*b*) comparable age and (*c*) comparable size, the latter tumour line having a reduced growth rate (Pearson, 1959*b*), were 1·75, 1·74 and 1·66 respectively. Total cells counted were between 14,600 and 18,000 in each group and the differences in mitotic proportions between the samples lay within the standard error. If the " B " cells were a post-mitotic degenerate form a substantial increase in the mitotic rate of " A " cells in the irradiated line would be necessary to account for the comparable overall rate.

The glassy-cell type of mixed carcinoma of the cervix, described by Glucksmann and Cherry (1956), appears to possess a nuclear norphology comparable to the " B " cells. These authors stated that this type of adenoacanthoma was comparatively refractory to irradiation. Gusberg *et al.* (1954 and 1956) suggested that the proportions of " A " and " B " cells in cervical cancer could be correlated with the clinical results from irradiation, a rise in the " B " cell proportion indicating a favourable result ; the assumption was made that the " B " cells were a degenerate form. The radiocurability of sarcoma 37 however was not significantly altered in its irradiated line, in spite of the considerable increase in " B " cells. It would appear therefore that cells of this nuclear morphology may not necessarily have comparable biological properties.

Measurements of DNA in control and irradiated line sarcoma 37 tumours (Pearson and Atkin, 1960) showed that the tetraploid mode in control tumours was replaced in the irradiated line by a mode corresponding to a triploid condition. Allowing for errors in the selection of classes in the DNA histograms and assuming the morphology of hexaploid and triploid nuclei to be comparable, the proportions of " A " and " B " cells in the two tumour lines are comparable to those of $4n$, $8n$ and $3n$, $6n$ cells estimated by DNA measurement. It would appear that a possible correlation exists between ploidy and nuclear morphology in this case.

It is of interest that in the sarcoma 37 experiments the serial application of irradiation produced permanent observable changes, not dependent on the continuation of the stress, which did not affect the radiosensitivity of the tumour. This suggests that a selection or change by irradiation of certain cell properties can occur without affecting the fundamental radiosensitive features directly concerned with cell reproducibility or viability.

### SUMMARY

1. Histological and cytological comparisons have been made on parent and serially irradiated lines of sarcoma 37 and two homologous tumours in RIII strain inbred mice.

2. The two homologous tumours showed no marked changes after serial irradiation.

3. Regions of increased anaplasia were apparent in the adenocarcinoma MV212 after serial irradiations.

4. Histological changes were apparent in the cortex of sarcoma 37 tumours subjected to sub-lethal doses at each irradiation stage.

5. In this same line a proportional increase occurred in certain cells having a characteristic nuclear morphology.

6. Both these changes appeared to be progressive during the establishment of the irradiated line and were not altered by sub-line passage without further irradiation treatment.

7. The significance of these changes is discussed in relation to radiosensitivity, cell degeneration and invasive properties.

8. A correlation is suggested between nuclear morphology and ploidy.

The expenses of this research were defrayed from a block grant by the British Empire Cancer Campaign.

### REFERENCES

CASPERSSON, T. AND SANTESSON, L.—(1942) *Acta radiol., Stockh.*, Suppl. 46.
GLUCKSMANN, A.—(1948) *Brit. J. Radiol.*, **21**, 559.
*Idem* AND CHERRY, C. P.—(1956) *Cancer*, **9**, 971.
GUSBERG, S. B., TOVELL, H. M. M., EMERSON, R. AND ALLINA, H.—(1954) *Amer. J. Obstet. Gynec.*, **68**, 1464.
*Idem*, TOVELL, H. M. M., LONG, M. AND HILL, J. C.—(1956) *Ann. N.Y. Acad. Sci.*, **63**, 1447.
MACKENZIE, D. H.—(1958) *Brit. J. Cancer*, **12**, 14.
PEARSON, A. E. G.—(1959*a*) *Ibid.*, **13**, 477.—(1959*b*) *Ibid.*, **13**, 699.
*Idem* AND ATKIN, N. B.—(1960) *Nature, Lond.*, **186**, 647.
SNELLMAN, B.—(1935) *Acta radiol., Stockh.*, **16**, 545.